# Thornwaldt cyst - treatment with diode laser

**DOI:** 10.5935/1808-8694.20130116

**Published:** 2015-10-08

**Authors:** Marco Antonio Thomas Caliman, Erika Mucciolo Cabernite, Juliana Tichauer Vieira, Diogo Carvalho Pasin, Denilson Storck Fomin

**Affiliations:** a MD. Otorhinolaryngologist.; b MD. Resident Physician in Otorhinolaryngology.; c MD. Resident Physician in Otorhinolaryngology, University of Santo Amaro - São Paulo.; d PhD in Medical Sciences - School of Medicine of Ribeirão Preto - University of São Paulo; Full Professor of Otolaryngology, University of Santo Amaro - São Paulo. University of Santo Amaro - UNISA.

**Keywords:** cysts, laser therapy, nasophary

## INTRODUCTION

The Thornwald cyst is a congenital cyst in the region of the pharyngeal bursa, formed by a communication between the notochord and the nasopharyngeal endoderm. It has an incidence of 3% in the adult population. Most cases are diagnosed during the second and third decades of life, with higher prevalence in males^1^.

Patients are usually asymptomatic, and they may present symptoms such as nasal obstruction, foreign body sensation, hearing loss, periodic halitosis with an unpleasant taste and nasopharyngeal discharge^1^, ^2^.

Since it is a benign lesion, asymptomatic cysts do not require treatment. Symptomatic cases can be operated by endonasal or transoral approach. Marsupialization is the procedure of choice to avoid recurrences^1^.

## CASE PRESENTATION

Female patient, 43 years old, had had constant and progressive nasal obstruction for the past 3 years, that worsened when laying down, accompanied by foreign body sensation in the posterior nasal region, postnasal dripping, halitosis, nasal itching and sneezing.

**Figure 1:**
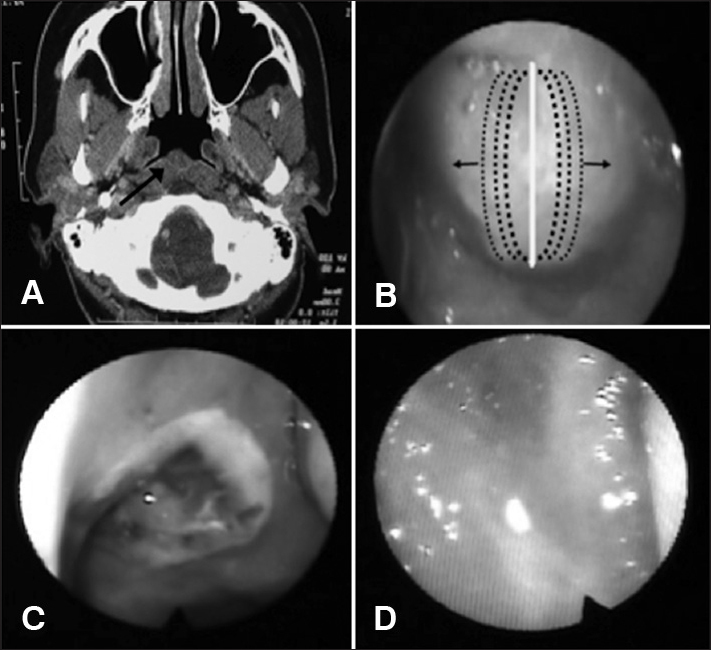
A: CT scan of the sinuses showing a lesion in the nasopharynx; B: Direction of the vaporization of the free edges of the cyst; C: Appearance of the surgical injury on the second day postoperatively; D: Three months postoperatively, normal looking mucosa without signs of recurrence.

Fiber optic nasal-laryngoscopy showed a yellowish cystic lesion with a smooth surface covered with mucosa in the posterosuperior nasopharynx, obstructing approximately 60% of its lumen. Computed tomography of the paranasal sinuses (Figure 1A) revealed a solid-looking lesion in the nasopharynx with a mild contrast detection capsule, and absence inside.

After detailed physical examination and imaging studies, we hypothesized the patient had a Thornwaldt cyst.

Due to the clinical symptoms, we indicated endoscopic surgical treatment of the lesion with the FOX^®^ (power 4.0 Watts) diode laser. We vaporized the anterior capsule of the cyst linearly and vertically, aspirated its contents (cytology) and removed small wall fragments (histology). After that, we progressively vaporized both free edges of the cyst in the lateral direction (Figure 1B), causing its retraction and exposure of the inner face of the rear wall. At the end of surgery, it was not necessary to place dressings or a splint.

In the immediate postoperative period, the patient had no complaints or bleeding. During the postoperative period, the patient remained asymptomatic and the surgical lesion showed rapid evolution without crusting, synechia or signs of recurrence (Figure 1C-D).

## DISCUSSION

Since the Thornwaldt cyst is a rare disease and basically asymptomatic, it is not often diagnosed. Thus, it is worth noting that the frequent use of endoscopy, such as nasopharyngoscopy, has been increasing the number of diseases diagnosed^3^.

The patient does not fit the classic epidemiological profile of the disease; however, the clinical history, physical examination and imaging studies were suggestive of a Thornwald cyst, which was confirmed by pathology.

Established treatment is drainage and marsupialization of the cyst, performed by transoral (by palate elevation) or transpalatine approach for larger cysts. It can be performed by the cold approach or use of electrocautery, which decreases the chance of bleeding; however, it uses high temperatures, damaging healthy tissues that permeate the cyst^3^, ^4^.

We chose to use the diode laser, which is characterized by a small diameter fiber, flexible and easy to handle. The method allows for endonasal treatment, which ensures good visualization of the lesion and surrounding structures, making the procedure safer and efficient^5^.

The area of thermal necrosis is only 0.5 mm, resulting in little injury to adjacent tissues, improving healing and reducing postoperative pain. It provides adequate hemostasis and control of tissue binding, facilitating the procedure and reducing its complications.

The main disadvantage of using laser is its high cost^5^. The disadvantages of the endonasal procedure are represented by its anatomical changes, such as septal deviations and turbinate hypertrophy, which often prevent adequate access to the posterior nasopharynx. In such cases, the corrections can be made in the same approach.

## FINAL REMARKS

The use of the diode laser to treat the Thornwaldt cyst proved, in our case, to be a safe and easy-to-perform procedure, with good postoperative results, making it a possible alternative for the treatment of these lesions.
